# P-165. Awareness of Rheumatic Heart Disease (RHD) and Treatment of Strep Throat in Kisoro and Gulu Districts, Uganda

**DOI:** 10.1093/ofid/ofaf695.389

**Published:** 2026-01-11

**Authors:** Alexandra C Hill-Ricciuti, Milton Aguyo, Ronald Wanyama, Felix Bongomin, Kevin Dieckhaus

**Affiliations:** University of Connecticut School of Medicine, Torrington, CT; Gulu University, Gulu, Gulu, Uganda; Gulu University, Gulu, Gulu, Uganda; Gulu University, Uganda, Gulu, Uganda; UConn Health, Southington, Connecticut

## Abstract

**Background:**

RHD continues to create significant morbidity and mortality in low- and middle-income countries, despite availability of penicillin to treat Group A streptococcal (GAS) infections. Understanding approaches to pharyngitis management, including ‘traditional’ non-allopathic methods (e.g., local tonsillectomy) and RHD awareness may lead to targeted interventions to eliminate RHD.

**Methods:**

Parents of children < 16 years of age, recruited from health clinics in Gulu and Kisoro Districts of Uganda, were administered an oral survey designed to evaluate knowledge, attitudes, barriers, and practices of pharyngitis treatment, including use of traditional medicine. Chi-square was used to examine associations between baseline characteristics (e.g., education) and responses to selected questions.

**Results:**

A total of 300 parents (n=150 Gulu, n=150 Kisoro) were recruited from July-August 2024. Most (86%) had heard of strep throat, but few (21%) knew it was caused by bacteria; 70% identified appropriate treatment. Many agreed untreated pharyngitis could lead to heart conditions (56%) or organ dysfunction (46%), but very few had heard of RHD (34%). Most disagreed (77%) local tonsillectomies were effective methods of treating sore throats, but most (60%) knew someone who had been treated with one. Most (58%) agreed allopathic health providers treat those who have sought local tonsillectomy poorly. Lower education was associated with decreased knowledge of appropriate pharyngitis treatment (p=0.04), but not with decreased awareness of RHD (p=0.20).

**Conclusion:**

Among parents in Gulu and Kisoro Districts, we identified low knowledge of proper treatment for strep throat, low awareness of severe sequelae of untreated pharyngitis (e.g., heart disease), and low awareness of RHD overall. We also found high usage of traditional medicine for pharyngitis and belief that allopathic providers treat those using traditional medicine poorly, which may represent treatment barriers. These data can inform community level primary prevention strategies. Future studies should address pharyngitis treatment and RHD diagnosis practices among healthcare providers in these districts.

**Disclosures:**

Alexandra C. Hill-Ricciuti, MPH, Bristol Myers Squibb: former employee|Bristol Myers Squibb: Stocks/Bonds (Public Company)

